# Vascular Contributions to Healthy Aging and Dementia

**DOI:** 10.14336/AD.2023.1719

**Published:** 2024-08-01

**Authors:** Yulin Ge

**Affiliations:** Department of Radiology, NYU Grossman School of Medicine, New York, NY, USA.

**Keywords:** aging, healthy, dementia, vascular, AD-related dementias, cerebral small vessel disease

## Abstract

Vascular pathologies are among the most common contributors to neurodegenerative changes across the spectrum of normal aging to dementia. Cerebral small vessel disease (SVD) encompasses a wide range of conditions affecting capillaries, small arteries, and arterioles, as well as perivascular spaces and fluid dynamics in the brain, playing a significant role in vascular contributions to cognitive impairment and dementia (VCID). These factors can accelerate the progression of SVD and neuronal degeneration. Since aging is the primary risk factor for Alzheimer’s disease (AD) and AD-related dementias (ADRD), this Research Topic aims to gather recent research to better understand vascular contributions to healthy aging and age-related cognitive impairment. Other risk factors include diabetes, lifestyle factors, high cholesterol, vascular inflammation, and immune remodeling, all of which can accelerate cognitive dysfunction progression. This special issue includes a total of 21 articles comprising Reviews, Perspectives, and Original Research articles. The articles cover various technical and biological aspects related to recent progress in aging and dementia research. We aim to promote research exchange across different fields, including imaging, VCID, molecular biology, neuroinflammation, and immunology. Most papers in this special issue focus on understanding the disease mechanisms of AD/ADRD and developing new therapeutic strategies.

## Cerebral small vessel changes in healthy aging and dementia

Cerebral small vessel disease (SVD) refers to a condition that affects the small blood vessels in the brain and is considered the main cause of white matter hyperintensities (WMHs). WMHs are quite common among older adults and are often found incidentally in MRI studies (e.g., FLAIR). Initially, SVD may not cause noticeable symptoms, but as it progresses, it can contribute to cognitive decline, particularly affecting executive function, information processing speed, and attention. The disease mechanisms involved in cerebral SVD may be associated with ischemic hypoperfusion, vascular inflammation, oxidative stress, and disruption of the blood-brain barrier (BBB) during normal and abnormal aging processes. Despite ongoing research, much of the intricacies underlying SVD and its subsequent outcomes remain inadequately understood. Consequently, the absence of targeted therapeutic interventions is not unexpected. The *in vivo* technical complexity of directly detecting and quantifying SVD presents a significant hurdle, impeding progress in elucidating its pathogenesis and the connections linking cerebral SVD to clinical manifestations like cognitive decline and dementia.

Although classic views of SVD from histopathology studies involve damage to the walls and narrowing of the small arteries and veins in the brain, *in vivo* imaging can only reveal WMHs, not their root cause of SVD. In this issue, Sun et al[1] demonstrated that age-related small-artery tortuosity changes can be detected using ferumoxytol-enhanced 7T MRI in healthy adult subjects. The corkscrew-like tortuous changes are mainly found in small medullary arteries ranging from 50 to 300 μm in diameter, providing blood supply to deep WM. The twisted small arterial changes can lead to reduced blood flow to brain tissue, which in turn can cause damage (e.g., WMHs) over time. This study represents the first direct visualization of small vessel (<300 μm) tortuous changes in live brains using ferumoxytol, an ultrasmall superparamagnetic iron oxide (USPIO), in combination with high-resolution T2*-weighted 7T MRI, leveraging its blooming effects and high susceptibility contrast. They have shown that age and BMI correlate significantly with both tortuous artery count and tortuosity indices. Clinical evaluation of vessel tortuosity at this micro-level is essential for the early detection of vascular risks and obtaining deeper insights into the vascular factors involved in cerebral SVD among the elderly. SVD plays a significant role in both Alzheimer's disease (AD) and vascular dementia, where reduced blood flow to parts of the brain deprives them of oxygen and nutrients, leading to cognitive impairments.

In another original research article, Allison et al.[2] investigated the association of arterial stiffness index (ASI) and brain structure in the dataset of UK Biobank. They observed significant negative association between baseline ASI and gray matter volume (p<0.001) 10-years post-baseline. ASI was measured using the PulseTrace PCA2 (Field-ID 21021) that can generate finger photoplethysmography from the end of the index finger to obtain the pulse waveform during a 10- to 15-s measurement using an infrared sensor and is a simple and quick approach for assessing arterial compliance. Despite ASI was measured peripherally, they identified a significant effect of baseline ASI on WMH volume at the 10-year end point after correcting for covariates of sex, age, and time between visits. These two studies imply that both arterial morphology and function changes can be potentially monitored to assess age-related trajectory of brain aging into older adulthood. It is worth noting that arterial stiffness either in small vessels or large vessels may lead to formation for twisted morphological changes [3].

Using arterial spin labeling (ASL) MRI, Zhou et al.[4] assessed the temporal relationship between reductions in CBF in the temporopariental region and temporal GM volume in a dataset with longitudinal design. They have shown that hypoperfusion in the temporal pole in AD patients, compared to normal controls or those with mild cognitive impairment (MCI), may be an early event driving its atrophy. Such reduced CBF is also thought to be caused by SVD. Additionally, for dementia related to Parkinson’s disease (PD), Ma et al.[5] suggested that instead of a single biomarker, combined biomarkers are more appropriate for improved diagnostic accuracy.

Mooldijk and Ikram[6], (needs update) in their review article, outlined the impact of cerebral SVD on cognitive decline and dementia, alongside an evaluation of current markers and their respective strengths and limitations. While MRI and transcranial Doppler sonography are commonly employed to identify vascular abnormalities, these imaging techniques may not fully capture all aspects of cerebral SVD. Considering the anatomical and physiological similarities between retinal vasculature and brain vasculature, recently retinal vascular imaging emerges as a promising avenue for assessing cerebral vessel integrity within the central nervous system (CNS). However, innovative techniques that allow direct visualization and quantification of cerebral small vessel morphology and functionality are crucial in early diagnosis and development of preventive therapeutic strategies. For example, Hoyer-Kimura et al.[7] used glycosylated Angiotensin-1-7 Mas receptor agonist PNA5 to reverse cognitive deficits, reduce ROS production, and inhibit inflammatory cytokines in their preclinical mouse model of VCID induced by chronic heart failure.

Li et al.[8], in a Perspectives article, elucidated the involvement of N6-methyladenosine (m6A) in vascular aging and associated disorders. m6A stands as the predominant modification found in eukaryotic RNAs, characterized by its reversible nature. The interrelationship of m6A modification with critical pathways involved in vascular aging links molecular insights to clinical implications, emphasizing its significance in comprehending disease progression. In another review article, Liu et al.[9] overviewed current research on ion transporters and channels at the BBB to address how the key ion transporters' expression and activity altered in AD and ADRD and whether these changes contribute to BBB dysfunction and disease progression. Dysfunction in Na^+^, K^+^, Ca^2+^, and Cl^-^ channels and transporters leads to osmotic cell swelling, activation of proinflammatory mediators, disruption of tight junction proteins, a common phenomenon in acute brain injuries.

SVD and AD are interconnected through various mechanisms involving the perivascular space (PVS), a key component of glymphatic system. In this issue, Sun et al.[1] demonstrated that there is an age-related increase in the number of tortuous medullary arteries found in the dilated PVS. Although further studies are needed to confirm this observation, SVD can exacerbate AD pathology by impairing blood flow, compromising the BBB, and disrupting the clearance of amyloid-beta through the PVS. Measuring flow or its disruption in the PVS clinically in patients is technically challenging. Recently, a novel method called diffusion tensor image analysis along the perivascular space (DTI-ALPS) has been used to assess the brain fluid dynamic system by measuring the motion of water molecules in the direction of the PVS. However, its reliability and reproducibility are still unknown. Liu et al. [10] conducted a multi-site, cross-vendor study for test-retest validation of this technique in 50 subjects. They demonstrated relatively strong inter-scanner reproducibility (ICC = 0.77 to 0.95, P < 0.001), inter-rater reliability (ICC = 0.96 to 1, P < 0.001), and test-retest repeatability (ICC = 0.89 to 0.95, P < 0.001), indicating that the ALPS index may serve as a viable biomarker for evaluating glymphatic system function *in vivo*. However, the value of DTI-ALPS in assessing differentiating AD from healthy or MCI patients is still unclear.

## Exercise, Circardian Rhythm, and Age-related Cognitive Decline.

Research indicates that AD pathology in people with increased cardiovascular risk factors can initiate decades before cognitive symptoms appear. Thus, preventive measures should be considered early in cognitively normal older adults or those with MCI, an early stage of AD. Strategies aimed at reducing arterial stiffness could enhance cerebrovascular function and lower the risk of AD. Previous studies have demonstrated that aerobic exercise training can enhance cardiovascular function and reduce central arterial stiffness. In this issue, Tomoto and Zhang [11] reviewed the impact of aerobic exercise training in older adults on vascular aging and function. Multiple key points have been summarized in their review article. First, the stiffening of large central elastic arteries is associated with advanced age. The elastic properties of central arteries (e.g., carotid arteries), due to the Windkessel effect, shield vital organs such as the brain and kidneys from potentially harmful excessive arterial pulsation, while maintaining efficient tissue perfusion. A mediation analysis indicates that carotid stiffness has direct and indirect contributions to number of WMHs mediated by the reduced CBF. It seems that the age-related changes of arterial morphology (e.g. tortuous) and function (e.g. stiffness) in both large[3] and small[1] arteries can potentially lead to increased number of WMHs. Second, arterial stiffness can be measured with cerebrovascular reactivity (CVR) using ultrasonography, transcranial Doppler (TCD), and hypercapnia MRI. Previous studies indicate that increases in CVR of carotid arteries were able to predict the onset of clinical AD independent of alterations of cerebral metabolism[12]. As large cerebral arteries like the ICAs are key contributors to overall CVR, these measurements offer insights into whether age-related increases in CVR are mainly due to vasoconstriction of smaller downstream cerebral arterioles and capillaries. There is a growing recognition that cerebral hypoperfusion, heightened CVR, and central arterial stiffening are emerging risk factors for clinical AD or VCID. Additionally, the CVR measurements of small arteries may indicate the levels of neuronal response or the integrity of neurovascular coupling (NVC). Third, despite that several studies have shown that aerobic exercise training reduces central arterial stiffness mainly through the modulation of arterial smooth muscle tone depending on nitric oxide bioavailability, the effects of aerobic exercise training on improving cerebrovascular function and cognitive performance in older adults are inconclusive. It is conceivable that exercise could diminish central arterial stiffness and arterial pulsation and enhance cerebral endothelial function, which may subsequently reduce CVR and elevate CBF[13, 14]. Tomoto and Zhang [11] also emphasized that the salutary effects of aerobic exercise training on brain health are likely to be multifactorial and go beyond those of aforementioned changes in cerebral hemodynamics.

On a similar topic, Tarnas et al. [15] discussed the effects of pilates training on cardiorespiratory functions in medical conditions. Pilates training has a positive impact on a broad spectrum of indicators of cardiorespiratory functions and significantly improves the quality of life, which may contribute to long-term behavior changes in patients related to overall physical activity. Their study will help to better understand the mechanisms linking cardiorespiratory functions with pilates training.

Circadian rhythms, governed by intrinsic biological clocks, are the body’s natural 24-hour rhythm that regulates physiological and pathophysiological processes. Recently, research has highlighted a two-way relationship between circadian rhythms and numerous neurological disorders. In their review, Huang et al.[16] presented a summary of the existing clinical and experimental proof concerning the impact of circadian regulation and why disruptions to circadian rhythms are a common feature of several neurodegenerative disorders including AD. These disruptions can lead to abnormal behaviors, physiological, and biochemical activities such as disrupted sleep-wake cycles, reduced hormone release and antioxidant production as seen in the elderly.

## Diabetes, obesity and AD pathology

Type-2 diabetes mellitus (T2DM) is closely associated with an increased risk of cognitive impairment through various biological pathways that include microvascular damage, hyperglycemia, insulin resistance, and inflammation. Diabetes can cause microvascular complications that lead to systematic vascular diseases including cerebral SVD (i.e., WMHs) and stroke, which are directly associated with VCID. In addition, impaired insulin signaling in the brain can affect cognitive functions, as insulin is involved in synaptic plasticity that is important for modulating brain networks and memory formation. Rhea et al [17], in their review article, have outlined the current state of research on brain insulin resistance (BIR) and cognitive decline. BIR is defined as an inadequate response by brain cells, including cerebral vasculature, to insulin. Causes of BIR include limited bioactive insulin in the CNS, reduced expression of insulin receptors (INSR) on cell surfaces, shifts in INSR isoform expression, and impaired downstream signaling from INSR binding. Although research focusing on understanding the mechanisms linking BIR and cognitive impairment is still ongoing, their review suggests that BIR is a plausible therapeutic target for the prevention of cognitive decline and dementia due to AD.

In addition, diabetes and obesity have a bidirectional and multifaceted relationship, with each significantly influencing the other's development and progression. Both share pathophysiological pathways such as oxidative stress, mitochondrial dysfunction, and endoplasmic reticulum stress. Excess body weight, particularly visceral fat, releases free fatty acids, pro-inflammatory cytokines, and adipokines, contributing to insulin resistance and cognitive impairment. A pilot study by Dolatshahi et al.[18] found that midlife (age: 51.27±6.12 years) obesity and excess adiposity are risk factors for AD. Higher visceral and subcutaneous fat ratios and PiB SUVRs (i.e., amyloid depositions) in the right precuneus cortex were linked to lower cortical thickness in AD-signature areas, considering age and sex. Additionally, higher BMI and insulin resistance were associated with lower cortical thickness in the bilateral temporal poles.

Another work from the same group by Wang et al.[19] examined the link between the WM connectome, obesity, and AD using edge-density imaging (EDI), a tractography-based method that characterizes the anatomical embedding of tractography connections. EDI maps the anatomical routes that pairs of gray matter regions use to communicate. By analyzing the average distribution of these routes, EDI assesses the structural connectivity pathways in the brain from ADNI dataset that included 30 participants known to convert from normal cognition or mild-cognitive impairment to AD within a minimum of 24 months of follow up as well as 30 non-converters. They found the edge-density-rich periventricular, commissural, and projection fibers were among the most important WM tracts linking BMI to FA and EDI from DTI data. WM fibers significantly contributing to the regression model related to BMI overlapped with those predicting conversion to AD, particularly in the frontopontine, corticostriatal, and optic radiation pathways. These fiber tracts are involved in the brain's reward network and hedonic food intake, creating a positive feedback loop between obesity and WM disconnection, which exacerbates further WM damage and abnormal eating behaviors. This study suggests that WM mapping with EDI enables the identification of abnormal connectome features implicated in both obesity and conversion to AD.

## Aging-induced cognitive decline: the role of neuroinflammation and immune remodeling

Aging is associated with changes in the immune system and neurovascular inflammation. AD is marked by a gradual decline in cognitive function and neuronal loss with some evidence highlighting the crucial role of glial cells—particularly microglia and astrocytes—in the onset and development of the disease. Deng et al.[20] reviewed these evidences showing that activated resident microglia enhance the transformation of resting astrocytes into reactive astrocytes, which promotes neurodegeneration during the progression of AD. These inflammatory responses involving neurons include "eat me" and "don’t eat me" signals, as well as processes like Aβ seeding, propagation, clearance, synapse loss, synaptic pruning, remyelination, and demyelination. More evidence from clean and well controlled experimental studies is needed to determine the specific role of the crosstalk between microglia and astrocytes in the aging process. Finally, they reviewed pharmacological and non-pharmacological therapies targeting microglia and astrocytes in AD.

In AD, hyperactivated astrocytes, the primary non-dividing glial cells in the central nervous system (CNS), often lead to neuroinflammation and cognitive impairments. The P2Y1 receptor (P2Y1R) has been implicated in AD pathogenesis. Luo et al[21]. investigated the impact of astrocytic P2Y1R on AD progression and its potential as a therapeutic target. They utilized P2Y1R knockout (KO) AD mice and AD mice with astrocyte-specific P2Y1R gene knockdown via short hairpin RNAs (shRNAs) delivered by an adeno-associated virus vector. Their findings demonstrated that P2Y1R inhibition reduced amyloid-beta accumulation, neuroinflammation, blood-brain barrier dysfunction, and cognitive impairment in AD mice. Additionally, decreased IL-6 gene expression in astrocytes confirmed reduced neuroinflammation, which correlated with improved blood-brain barrier integrity and cognitive function. These results highlight that astrocytic P2Y1R accelerates AD pathology through neuroinflammation, suggesting that silencing astrocytic P2Y1R could be a novel therapeutic target for AD.

One study in this issue has focused on bacteria and aging to unravel the interplay for healthy longevity and dementia. Liu and Sun[22] have highlighted lactic acid bacteria (LAB) as promising dietary options for promoting healthy aging and improving quality of life in the elderly. LAB, a prominent component of the gut microbiota and often used in food fermentation and probiotic supplements[23], may offer anti-aging benefits by restoring gut balance, enhancing antioxidant potential, and supporting cognitive health. However, current evidence is preliminary, requiring more human trials and detailed mechanistic studies. Future research should identify LAB strains with significant anti-aging effects and explore their biological mechanisms.

Additionally, Soraci et al.[24] [needs update] summarized the current knowledge about neuroinflammaging with a particular focus on epigenetic mechanisms underlying the onset and progression of neuroinflammatory cascades in the CNS. Inflammaging was originally defined by Franceschi et al.[25] and is a persistent, low-level, sterile inflammatory process that enhances the brain's inflammatory response and contributes to the development of neurodegenerative diseases associated with aging. Although neuroinflammaging level is now recognized as a risk factor for the development of the most common age-related diseases, the exact mechanisms need further thorough evaluation. Along the same direction, Dou et al.[26] outlined age-related changes in the gut microbiota that can shape the immune and inflammatory responses. They described the concept of immunonutrion, which has been regarded as a new strategy for disease prevention and management. Both quantitative and qualitative studies on this topic are reviewed, indicating immunonutrition-based interventions may play a role in healthy aging.

Autophagy is a self-degradation process in cells, utilizing autophagosomes to break down and clear dysfunctional organelles and recycle cellular components. Wang et al.[27] explored the mechanisms of autophagy in different diseases and highlighted its role in disease development and progression. Their review is expected to help shape new therapeutic strategies and offer different perspectives for developing innovative treatments as autophagy can be a positive and negative force for maintaining good health and dealing with various disease conditions caused by pathogenic infections.

## Conclusion

In summary, this is a pivotal moment for aging and dementia research, spurred by the urgent demand as more individuals reach old age. This Research Topic aspires to offer an extensive compilation of recent progress spanning a wide array of cross-disciplinary subjects on healthy aging and dementia.
